# Solid-Phase Extraction Strategies to Surmount Body Fluid Sample Complexity in High-Throughput Mass Spectrometry-Based Proteomics

**DOI:** 10.1155/2015/250131

**Published:** 2015-01-27

**Authors:** Marco R. Bladergroen, Yuri E. M. van der Burgt

**Affiliations:** Leiden University Medical Center (LUMC), Center for Proteomics and Metabolomics, P.O. Box 9600, 2300 RC Leiden, Netherlands

## Abstract

For large-scale and standardized applications in mass spectrometry- (MS-) based proteomics automation of each step is essential. Here we present high-throughput sample preparation solutions for balancing the speed of current MS-acquisitions and the time needed for analytical workup of body fluids. The discussed workflows reduce body fluid sample complexity and apply for both bottom-up proteomics experiments and top-down protein characterization approaches. Various sample preparation methods that involve solid-phase extraction (SPE) including affinity enrichment strategies have been automated. Obtained peptide and protein fractions can be mass analyzed by direct infusion into an electrospray ionization (ESI) source or by means of matrix-assisted laser desorption ionization (MALDI) without further need of time-consuming liquid chromatography (LC) separations.

## 1. Introduction

**Figure 1:**
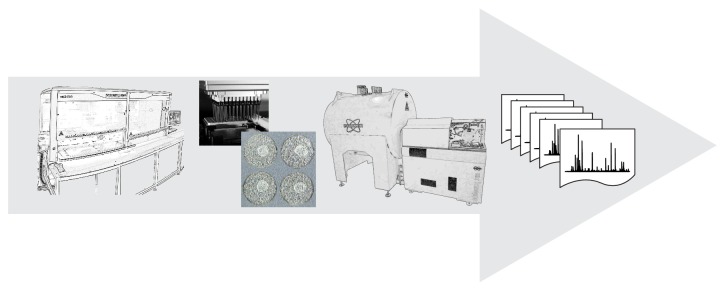
Peptides and proteins are isolated from body fluids using a fully automated solid-phase extraction protocol based on functionalized magnetic beads. All samples are processed in 96-well plate format with a 96-channel pipetting head, increasing throughput up to 1000 samples per 24 hours. After spotting onto a matrix-assisted laser desorption ionization (MALDI) target plate peptide and protein profiles are acquired on an ultrahigh resolution Fourier transform ion cyclotron resonance (FTICR) system.

Sample preparation of biological specimens is an essential part in any mass spectrometry- (MS-) based proteomics experiment since body fluids as well as tissue sections are highly complex in terms of variety in components and their concentrations. A wide and diverse range of sample preparation strategies has been developed that provide sample (protein) fractions for further identification and quantification by MS. The preferred route for sample prep obviously relates to the actual research question taking into account that any bias should be avoided. For comprehensive coverage of the proteome the isolation of as many peptides and/or proteins as possible is required [1]. However, for high-throughput (HTP) purposes the capture of a small set of peptides can be sufficient in order to answer a specific question. In our laboratory, we have developed fully automated sample preparation pipelines that allow MS-profiling of body fluids at HTP (500–1000 samples per 24 hours) and have been applied in clinical studies based on biobanked samples [2–4]. Initially, most of these so-called peptide and protein profiles were acquired on matrix-assisted laser desorption ionization (MALDI) time-of-flight (TOF) mass spectrometers [5, 6]. Although this combination still is extremely powerful in terms of costs and analysis time, we have recently shifted the MS-workflow to an ultrahigh resolution Fourier transform ion cyclotron resonance (FTICR) system in combination with MALDI (see Figure 1) [7, 8]. The low part-per-million (ppm) mass accuracy of peptides and proteins observed between 1 and 9 kDa in such FTICR profiles facilitated sequence identifications while excellent case-control classification characteristics were conserved. Increasingly and in particular for case-control disease studies high-throughput analysis is required, that is, short measurement times or multiplexed analysis. HTP platforms that allow the analysis of large numbers of samples are needed in both a research setting and a hospital setting where doctors and patients demand fast answers. Efforts to optimize throughput parameters should therefore be considered during the design of a procedure. Furthermore, when performing quantification experiments it is important to reduce the number of handling steps prior to analysis, in order to obtain minimal loss of analyte, and to have a good estimation or, better, measurement of the efficiency of recovery.

Many proteomics studies are based on blood, serum, and/or plasma samples. It is generally assumed that blood reflects the state of the body. Blood samples are relatively easy to obtain and the concentration of protein/peptides in “blood-derived” samples is high (reference range for serum is 60–85 g·L^−1^ total protein). Although urine can be obtained even in a noninvasive way, it is much less used in proteomics studies. Disadvantages of urine are the presence of MS-hampering salts and a lower concentration of proteins and peptides compared to plasma and serum, namely, normal total protein concentrations of about 20 mg·L^−1^ in a standardized sample based on healthy individuals [9]. Moreover, proteins and peptides present in urine may not correlate with the state of the body and blood samples do, with the exception of, for example, kidney diseases [10, 11]. Alternatively, cerebrospinal fluid (CSF) is a relatively “clean” sample and can often be further used without extensive preparation steps. It can however not be easily obtained and if so only in small volumes. In this paper various sample preparation strategies for body fluid analysis are discussed. Previously, prefractionation techniques aiming for (clinical) MS-based proteomics have been overviewed extensively [12–18]. All workflows discussed in the current review are based on solid-phase extraction (SPE) and can be combined with HTP bottom-up as well as top-down proteomics approaches.

## 2. Body Fluid Sample Collection

Standardized body fluid sample collection and sample storage protocols are crucial for appropriate sample preparation. The aim is to time-snap the state of the sample or, in other words, to keep the composition and quality of the sample as close as possible to the time point at which the sample was taken. In the case of serum and plasma, important parameters are processing times (e.g., the clotting time of a blood sample), storage times, storage temperatures, and number of freeze-thaw cycles [19, 20]. For plasma samples additives such as heparin or citrate are needed to prevent clotting. It should be noted that the presence of heparin in a complex protein sample will influence chromatographic performance, as well as the anticoagulant ethylenediaminetetraacetic acid (EDTA). Consequently, these additives often need to be removed prior to analysis. Furthermore, it should be noted that clotting (or coagulation) factors are removed in serum samples (listed at http://en.wikipedia.org/wiki/Coagulation) and that additional proteins or peptides with affinity to these clotting factors can be removed undesirably. Another variable that is of great importance in sample preparation involves the consumables that are used for collection and processing. It is known that certain brands of plastic containers may release polymers or other interfering species [21]. For example, we have detected bovine serum albumin (BSA) from adhesive foils to close microtitration plates (MTPs) as a major interfering compound. Proteins and peptides may also adhere to tube walls giving rise to significant differences in protein composition between two aliquots of the same sample processed in different tubes [22, 23].

## 3. Solid-Phase Extraction of Proteins and Peptides

Solid-phase extraction (SPE) is widely used in MS-based proteomics as a sample preparation technique. With SPE compounds are isolated on basis of their chemical and physical properties which determine their distribution between a mobile liquid phase and a solid stationary phase. After binding of the molecules with the correct properties, the remaining compounds are washed away and the bound molecules are eluted from the solid phase by changing the mobile phase into the elution solvent. Whereas a chromatographic column for separating compounds by elution with a gradient is used multiple times, SPE material is usually disposed after each sample and no gradient is applied for elution (one-step elution) [26]. Thus in theory all compounds present in the sample are captured with chromatography, while with SPE only a certain group of analytes is isolated, depending on the solid phase. Therefore SPE is mainly used to clean up a sample and reduce sample complexity. For protein analysis with MS it is often used to remove salts and other impurities that might cause ion suppression. However, by carefully choosing the right SPE sorbent a higher selectivity can be achieved. This selectivity also means that in principle with SPE only a subfraction of the sample is analyzed, because not all compounds are captured, but only those compounds that match the binding capabilities of the sorbent. SPE material is available in various formats, including (micro-) columns, cartridges, plates, micropipette tips, and functionalized magnetic beads (MBs). The latter have several advantages over the other formats. First, their small size allows a higher concentration rate and therefore sensitivity due to their large binding surface area [27, 28]. Second, the use of MBs can be automated, providing a highly consistent high-throughput method [2]. Recently, an automated method for tip-based SPE has been developed [29]. However, a difficulty in manufacturing SPE tips is the degree of packing. Generally, SPE tips are not equally packed. Differences between tips become apparent when monitoring liquid handling performance in terms of back pressure upon wetting and elution of the tip. For this reason, the implementation of SPE tips on a robotic pipetting system is not trivial; that is, it is difficult to program a robot in such a way that each tip is sufficiently wetted and similar elution volumes are obtained. For example, the default mixing feature in the software running our robotic system results in inadequate washing due to the slow liquid flow in the tip packed with SPE material. To correct for this, a settling time is needed after the aspiration step as well as after the dispense step during the mixing cycle, similar to the procedure generally implemented for very viscous liquids. Examples of integrating SPE cartridges in an MS-based bottom-up workflow have been reported [30, 31].

The most commonly used SPE material in proteomics for the isolation (or fractionation) of proteins and peptides is reversed-phase and to a lesser extent ion-exchange material. For the isolation of glycosylated proteins and peptides normal phase including HILIC is preferred [32, 33]. A summary of SPE materials is given in Table 1, partially based on an earlier overview [24, 34]. Less commonly used SPE materials are for instance silica- or polystyrene-based [35, 36]. Recent advances in ion-exchange chromatography have been made with mixed-bed materials [37]. Mommen and coworkers combined weak anion-exchange (WAX) with strong cation-exchange (SCX) material [38]. In this way, they were able to perform a salt-free elution using a diluted formic acid solution to which DMSO was added, while optimizing the WAX/SCX ratio. Peptide recoveries could be improved in comparison to SCX alone with a WAX/SCX ratio of 4. This improved recovery could be explained by the so-called Donnan effect, as well as by the absence of salts (that are usually incompatible with MS) by using a pH block-gradient [39]. This Donnan effect also accounts for binding of proteins to newly developed core-shell nanoparticles. Core-shell nanoparticles are composed of a solid interface covered with a shell of charged polymers either as microgel or as densely grafted linear polymers (so-called polyelectrolyte brushes). The exact mechanism that is involved in the binding of proteins to these nanoparticles is described [40]. Nanoparticles have successfully been applied in the isolation of low-abundance proteins, as not only are electrostatic interactions important, but these nanoparticles also have molecular sieving properties, which prevent large high-abundant proteins such as albumin from being captured. Further advantageous properties of these nanoparticles are their concentrating capacity and large surface area and their protectiveness against enzymatic degradation and other environmental influences on the captured peptides [41].

Another specific example of solid-phase extraction of peptides and proteins involves the application of a solid-phase peptide library [42, 43]. Such a library allows for the enrichment of low-abundant proteins, while simultaneously reducing the relative concentration of abundant species, without needing protein depletion. In this approach a complex protein sample is exposed to a ligand library in large overloading conditions, resulting in a rapid saturation of each bead with affinity to an abundant protein and a remaining vast majority of the same protein in solution (i.e., unbound) [44, 45]. The beneficial aspect is that trace proteins will not saturate the corresponding partner beads but are captured in progressively increasing amounts. This process of equalization of the large range in protein concentrations or compression of the sample dynamic range is commercialized with the ProteoMiner protein enrichment kit. This combinatorial hexapeptide library has been applied in various MS-based proteomics studies (including at our own laboratory) and it was found that the large variation in protein concentrations was decreased in complex biological samples, thus resulting in a relative enrichment of medium- and/or low-abundant proteins [46–55]. Generally, the increment in the number of species detected was from twofold to fivefold compared with control, nontreated samples. This substantial increment in detection applies mostly to low-abundance proteins, considering that they could not be detected in the untreated samples, where they have been obviously present.

In our laboratory, we have implemented various SPE protocols (based on MBs) in a modular way on a liquid handling Hamilton robot [2]. In addition, for bottom-up proteomics purposes, a protein digestion protocol has been implemented on the same robotic platform allowing both in-gel or in-solution digestion. In the first case, the digestion protocol starts with several washing steps of the gel-plugs, followed by reduction/alkylation of the proteins and peptides and additional washing steps of the gel-plug, followed by incubation with a proteolytic enzyme at elevated temperatures and an acidification step to deactivate the enzyme. In the case of in-solution digestion, the same procedure holds with the exception of the additional washing steps. The digestion protocol can be continued with a peptide purification step by means of SPE, generally in the form of a reversed-phase C18-tip. On our robotic system, purification with C18-MBs has proven to be a powerful method. Moreover, cartridge-based SPE protocols for protein extraction were developed and automated on a Spark Symbiosis system [2].

## 4. Affinity Enrichment of Proteins and Peptides

**Figure 2:**
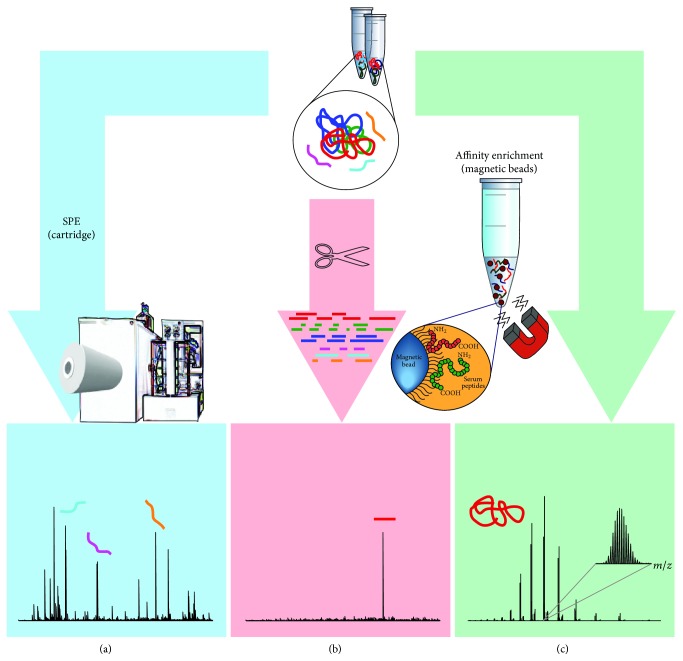
Starting with a complex body fluid sample a specific peptide or protein can be enriched by means of immunoaffinity capture. Whereas a subfraction of the sample is analyzed in an SPE approach, profiling compounds that match the binding capabilities of the sorbent (a), affinity enrichment results in “clean” mass spectra (proteolytic peptide in (b), intact protein in (c)).

Affinity enrichment is based on the reversible and noncovalent interaction of the protein or peptide of interest with naturally occurring (or synthetic analogues of) biomolecules, such as other proteins, lectins, nucleotides, or dyes. Affinity enrichment is a popular chromatographic method for the immunocapture of compounds in larger quantities, for instance, immunoglobulins (IgGs) with protA or protG. It is however also a convenient method for SPE of smaller sample volumes aiming for capture of a specific protein or peptide (see Figure 2). As presented in the previous section and in Table 1, various materials for affinity enrichment are available [56]. Moreover, immunocapture strategies can also be used for depletion of high-abundant proteins to allow access to the low-abundant ones (in-depth analysis) [57]. For this purpose, commercially available products make use of a mix of antibody-based affinity material, such as the Proteoprep 20 Plasma immunodepletion kit from Sigma, which removes the 20 most abundant proteins in a single purification step [58], or MARS from Agilent [59]. Qian and coworkers used the commercially available ProteomeLab IgY12 affinity column (Beckman Coulter, Fullerton, CA) in combination with a custom made Supermix column to separate more than 50 high- and medium-abundant proteins from the lower-abundant ones in a plasma sample [60]. In the case of enrichment strategies, in general more information about the analyte of investigation is already available and the research question has focused on a very specific group of proteins or even a single protein. An elegant example of immunoaffinity (IA) enrichment was shown by Halfinger and coworkers [61], which involved a thermal protein precipitation step, followed by liquid-liquid precipitation before IA chromatography to quantify the low-abundance heart failure marker N-terminal pro-B-type natriuretic peptide (NT-proBNP). This example showed a combined preparation method to deplete high-abundant proteins followed by concentration of a specific low-abundant protein through immunoaffinity. The simple and cost-effective method could be used to unmask low-abundant proteins in general. Finally, it should be noted that another important and widely used application of affinity purification concerns the isolation of phosphoproteins through metal-affinity SPE and that the isolation of glycoproteins or glycopeptides through lectin-affinity is a field of their own [62].

A combination of protein digestion and affinity enrichment SPE of a peptide is used in the so-called workflow of SISCAPA, which stands for Stable Isotope Standards with Capture by Anti-Peptide Antibodies. This targeted protein quantification approach was coined in 2004 by Anderson and coworkers [63] and comprises the isolation of a known (proteolytic) peptide (from a clinically relevant protein) by means of antibody coated SPE material after proteolysis of the total sample proteome. Where protein digestion was originally used in the discovery stage of MS-based proteomics studies to allow protein identifications, SISCAPA aims for absolute quantification of the analyte in a clinical setting. Such quantification relies on the use of external calibrators, such as a stable-isotope-labelled peptide (with identical amino acid sequence), in order to enable determination of the concentration of the released proteolytic peptide and thus the endogenous protein. For this purpose, precise and detailed knowledge on the extent and reproducibility of the digestion is crucial [64]. With regard to sensitivity and limit-of-detection, a number of IA techniques in combination with MS have been reviewed [65]. Although ELISA still outperforms MS-based techniques in terms of sensitivity, the potential of SISCAPA to allow quantification of low-abundant proteins in body fluids is generally acknowledged. A further increase in sensitivity is expected from improvements in MS equipment. Recently, the SISCAPA workflow has been automated by means of magnetic beads and concurrently improved sensitivity to the pg·mL^−1^ range by increasing the sample volumes [31, 66, 67]. Similar methodologies have been proposed, based on immunocapture at the protein level [65]. Here, the wider availability of antibodies against many proteins could be advantageous. It is further suggested that sensitivity improves with the number of so-called proofreading steps [68]. A proofreading step is regarded as a step in an assay that results in a stepwise decrease in the error fraction. We foresee that the combination of the two techniques mentioned by Ackermann and Berna will add extra proofreading steps (two different antibodies instead of one) and together with the improvements in SISCAPA workflows will increase sensitivity even more.

## 5. Conclusions

High-throughput analysis is required in particular for clinical samples when MS-based proteomics is applied to case/control disease studies. Sample preparation strategies are aimed at reducing sample complexity and improving quality of the measurements. The automation of protein purification methods has become crucial in order to balance the speed of MS-acquisition for both bottom-up and top-down proteomics analyses. Additional advantages of a robotized process are improved reproducibility and standardization of the workflow. Processes on a robotic platform should be implemented in a modular way to allow flexibility in choosing SPE materials and solvents. The selection of an SPE method should be based on the potential for automation. For exploratory as well as for screening studies, general solid phase materials such as RPC18 and SCX are widely used, whereas a targeted approach requires a specialized solid phase such as affinity SPE material. High-throughput strategies have been developed for SPE materials on various formats, including cartridges, micropipette tips, and functionalized magnetic beads (MBs).

## Figures and Tables

**Table 1:** Various sorbents used in protein SPE.

SPE type	Material	Usage

Reversed-phase	C3, C4, C8	Proteins
	C18	Peptides

Normal phase	ZIC-HILIC	Glycoproteins, glycopeptides
	TSKgel Amide-80	Proteins
	Waters HILIC	Proteins, peptides
	Cotton^*^	Glycopeptides, glycans after release from peptides

IEX	WCX, SCX	Proteins
	WAX, SAX	Proteins

Metal-chelating	Ti, Fe	Phosphopeptides
	Ga	Phosphopeptides
	Cu	(Phospho)peptides

Affinity	Lectin	Glycans, glycopeptides
	Boronic acid	Glycans, glycopeptides
	Blue dye	Albumin
	Protein A/G	Immunoglobulins
	Heparin	IgG
	RNA/DNA	Plasmids, DNA binding proteins
	Purine/pyrimidine derivatives	For example, ATP/GTP using enzymes
	Coenzymes	Coenzyme-dependent enzymes
	Vitamins	Vitamin binding proteins
	Antibodies	Proteins, peptides

For further information see [24] and references cited therein.

^*^Reference [25].
